# Artificial intelligence machine learning-driven outpatient appointment management: A qualitative study on acceptability

**DOI:** 10.1177/20552076251321016

**Published:** 2025-06-17

**Authors:** Kerry V. Wood, Daniel Frings, Chris Flood, Nicola Thomas

**Affiliations:** 1 Department of Psychology, 4914 London South Bank University, London, UK

**Keywords:** Appointment management, artificial intelligence, acceptability, digital health, technology adoption

## Abstract

**Introduction:**

Managing outpatient appointments is challenging, with missed appointments wasting capacity. Artificial Intelligence (AI) machine learning-driven automated reminders offer a solution, but their success relies on patient and staff engagement, highlighting the need for impact assessment.

**Objective:**

To investigate the acceptability of AI machine learning-driven appointment management for patients and staff, identifying barriers and facilitators.

**Methods:**

Semi-structured interviews with seven staff and twelve patients. Despite scheduling efforts and incentives, practical constraints limited the sample size and generalizability. Interviews were analysed separately using Thematic Analysis, with one researcher coding and categorizing data, followed by discussions to refine themes and validate quotes.

**Results:**

Five themes emerged. Patients: ethical concerns, AI understanding, reminder efficacy, user satisfaction, and usability. Staff: AI understanding and hesitancy, barriers and drivers, technology experiences, appointment management, and sustainability. Barriers included privacy concerns, limited interactivity, fragmented integration, and operational challenges. Facilitators were perceived prediction accuracy and reminder usefulness. Patients valued usability, convenience, and reminders but sought better interactivity and integration. Staff emphasized ethics, operations, and sustainability, with motivation linked to reduced DNAs. Both valued accuracy and reliability, highlighting the need for tailored strategies.

**Conclusions:**

This study explores patient and staff perceptions of AI in NHS appointment management. Despite high trust in data security, privacy concerns, inefficiencies, and limited interactivity hinder adoption. Accuracy and convenience drive engagement. Findings highlight the need for better integration, clarity, interactivity, and accessibility to enhance user experience and AI adoption in healthcare.

## Introduction

Managing outpatient appointments presents significant challenges for healthcare providers and patients alike, with missed appointments leading to lost treatments and wasted capacity. When patients fail to attend scheduled healthcare appointments without prior cancellation, these “no shows” or “DNAs” (“did not attend”) result in time slots that cannot be reallocated to other patients. This inefficiency frustrates healthcare clinicians and planners as it leads to lost time, decreased operational efficiency, and increased resource utilization.^1^ For patients, longer waiting times due to missed appointments contribute to increased dissatisfaction, reduced quality of care, and potentially worsened health outcomes for themselves and others needing timely appointments. In 2015, the UK National Health Service (NHS) estimated that missed appointments cost £912 m annually,^2^ with many appointments missed due to simple reasons such as forgetfulness.^3^

To reduce the “DNA” rate, outpatient services have employed various methods, including postal reminders, emails, and telephone reminders. However, these traditional approaches can be costly in terms of time and staff resources. Consequently, artificial intelligence (AI) machine learning technologies have gained prominence as innovative solutions to address healthcare inefficiencies.^4,5^ Such reminders, for instance, offer a cost-effective and scalable approach by targeting individuals most likely to miss appointments, leveraging predictive analytics to enhance resource optimization.^6^ Research has also highlighted AI's potential to automate routine administrative tasks, such as appointment scheduling, resulting in significant operational efficiencies.^7^

Given that mobile (cellular) telephone subscriptions match the global population,^8^ sending electronic text reminders is increasingly popular due to its low cost, scalability, simplicity, and public acceptability. Studies show that automated text reminders can reduce missed appointments by up to 28%, highlighting their impact on healthcare efficiency.^9^ Furthermore, the use of AI-driven reminders reduces the number of unnecessary messages, focusing resources where they are most needed, which can contribute to long-term cost savings for healthcare systems.^10^

When implementing new AI-driven applications in healthcare, it is crucial to consider their impact on patient experiences and staff interactions with the systems. Despite the growing adoption of AI technologies in healthcare, there remains a gap in understanding how these systems are perceived by end-users, particularly in the NHS, where unique structural and operational challenges exist.^11,12^ Existing studies have largely focused on technical feasibility or clinical outcomes, with limited attention to user acceptability or barriers to implementation. A key factor in this context is the acceptance of AI machine learning-driven interventions by staff and patients and their willingness to engage with these technologies.^13^

This study aimed to explore the experiences of patients and staff with an AI machine learning-driven appointment management system currently implemented in parts of the NHS. Specifically, it sought to address gaps in the literature by examining the acceptability of this system and its operational impact in a real-world healthcare setting. To date, no research has evaluated its acceptability. The focus was on assessing its acceptability and identifying barriers and facilitators that may influence the effectiveness of these services.

## Method

### Design

This study was a component of a broader mixed methods trial that assessed the accuracy, safety, effectiveness, value, and person-centredness of an AI machine learning-driven appointment management system, including a process evaluation. This paper specifically addresses only the qualitative findings on acceptability. We adopted a qualitative approach, using online semi-structured interviews with NHS patients and staff to explore the acceptability of the system.

### Interviews

The qualitative interview schedules were guided by the framework for examining patient attitudes regarding applications of AI in healthcare^14^ and the theoretical framework of healthcare intervention acceptability which proposes definitions and assessment tools for evaluations of the acceptability of new or existing interventions.^15^ In addition, the interview questions were discussed with the project team's Patient and Public Involvement (PPI) group. The interview schedule consisted of four sets of open-ended questions exploring predetermined themes including understanding of AI, AI hesitancy, motivations, and experiences. Prompts were used to glean more in-depth data (see Table 1).

**Table 1.** Interview questions for patients and staff.

*Understanding of AI*
What do you think is your level of experience with technology generally?
What do you know about artificial intelligence (AI)?
What are your thoughts on using AI in healthcare? In appointment management?
What do you know about this (appointment management) technology?
*AI hesitancy*
How accurate do you think this technology would be at managing appointments?
Do you think you can trust this technology in terms of being able to tailor appointments to your/patients’ specific situation?
Do you feel this technology will be able to protect your/patient information?
*Motivations*
How far do you feel that this technology is flexible when managing appointments?
Have you been receiving sufficient reminders of your appointments? Or too many? (patient question only)
*Experiences*
Have you had previous interactions or experiences with AI in healthcare?
What is your experience of using this technology?
Have you noticed any changes in your/patient appointment scheduling since the technology was introduced?
How well has this technology integrated with other appointment scheduling services?
Could you foresee any potential problems using this technology?
Could you see any value in future and more widespread uses of this technology?
How satisfied are you with the technology? Could anything be improved further?

### PPI group

A PPI group was convened at the start of the evaluation period. Following development of a PPI group role description, an advert for PPI group members was distributed among renal, cancer, and ophthalmology charities. Eight people with diverse experience of renal, oncology, and/or ophthalmology care participated in the PPI group throughout, and at the end of the project seven were still part of the group. Ten PPI group meetings were held. The first meeting explained the scope of the evaluation and the different evaluation questions. The PPI group subsequently advised on issues surrounding access to identifiable patient data (requirements of the Confidentiality Advisory Group) and discussed and agreed patient survey items and interview questions.

### Study setting 

The study was set in a central England NHS Trust outpatient's department, specifically with staff and patients in specialties involved in the trial of the AI machine learning-driven appointment management system (renal, oncology, and ophthalmology outpatients). These specialties were chosen because they involve a higher number of patients with linked appointments across specialties, which increases the likelihood of appointment errors. This made them suitable for testing the AI system's capabilities in managing complex scheduling scenarios. Additionally, these specialties represent busy outpatient departments with diverse patient demographics, providing a representative sample for evaluating the system's effectiveness. However, it is acknowledged that focusing on these specific specialties may limit the generalizability of findings, as the unique workflows and patient populations in these departments may not fully reflect those in other specialties or healthcare settings.

### Recruitment of staff

The recruitment of clinical and administrative staff was facilitated by the hospital-based AI system project manager, who invited (via email) individuals involved in trialling the AI system within renal, oncology, and ophthalmology departments to participate. Staff were provided with an information sheet, and those who agreed to take part had their contact details shared with the university research team, who scheduled online interviews via MS Teams. To encourage participation, staff were offered time out during their working day; however, due to time pressures associated with their roles, only seven of the 10 contacted were able to attend. This limited sample size may restrict the generalizability of the findings, as the experiences and perspectives of staff who were unable to participate could differ, potentially resulting in an incomplete representation of the broader staff population involved in using the AI system.

### Recruitment of patients

As part of the larger study, patients completed a survey about the AI system and as part of this were asked if they would be willing to be contacted by the research team for interview. If willing, they provided their telephone/email address for the research team to contact them to take consent and organize a suitable time for telephone interview.

Patient inclusion criteria:

1. Patients receiving secondary care (outpatients) at intervention sites.
2. Aged 18 years or over.
3. Within one of the following care streams: ophthalmology (diabetic eye), renal (advanced kidney care and renal replacement therapy), and oncology (colorectal).

The research team attempted to contact (via email or telephone) 26 patients who had provided contact details on the survey. Of these patients, 10 were no longer willing to take part (four changed their minds, two felt too unwell, and four gave no reason), while another four did not respond to telephone calls or emails. Ultimately, 12 patient interviews were scheduled and conducted over the telephone with only the researcher and interviewee present. Efforts were made to maximize participation, such as providing a financial incentive and accommodating patients’ schedules. However, the relatively small sample size may limit the generalizability of the findings, as the perspectives of patients who declined or were unable to participate might differ from those who completed the interviews. This highlights the potential for selection bias, particularly if those who declined faced barriers or held views that were not captured in the data.

Patients who completed the interviews were given a £10 voucher as a token of appreciation for their time and contribution to the study. The decision to offer this compensation was guided by ethical considerations to acknowledge the effort and time taken by participants, particularly given that the interviews required their active engagement and took place during their personal time. The amount was set at a modest level to avoid undue influence on participation decisions, ensuring that the incentive did not coerce or bias individuals into taking part.

All participants were asked to sign a consent form and were informed about their rights to confidentiality and that they could withdraw from the study at any time. Interviews lasted 20–45 minutes, were audio recorded, and transcribed verbatim. Once researchers (NT and KVW) felt thematic saturation was reached, no new patient interviews were scheduled.

### Interview team

Interviews were conducted in 2023 by two female experienced qualitative researchers (KVW and NT). NT is a professor of kidney care and a registered nurse; KVW is a registered health psychologist with a PhD. There was no relationship between interviewers and interviewees and interviewees were told the researchers were conducting an independent evaluation and were not linked to the AI technology.

### Data analyses

Thematic analysis^16^ was used to identify common patterns and trends, separately for patient and staff interviews. One researcher, KVW, familiarized herself with the transcripts by reading and re-reading them, and used NVIVO software to code and organize data into meaningful themes and sub-themes. The coding process involved both deductive and inductive approaches: deductive codes were informed by the research questions, while inductive codes emerged directly from the data. To ensure rigour, the initial coding framework was reviewed and refined through iterative discussions between KVW and NT.

Themes were validated through a collaborative process where both researchers compared coded data to ensure consistency and relevance, and discrepancies were resolved through discussion. Key quotes were cross-checked against themes to ensure that they accurately reflected the underlying data. This iterative approach helped ensure that the final themes were both comprehensive and representative of the participants’ experiences. Regular discussions provided an additional layer of validation, enhancing the reliability of the analysis.

Thematic saturation was reached for patient interviews, as no new themes or sub-themes emerged from the analysis of later transcripts. However, thematic saturation was not reached for staff interviews due to the limited number of staff available to participate. This limitation was compounded by the fact that only a small number of staff were directly involved in the trialling of the AI system and therefore suitable for interview. While efforts were made to capture diverse staff perspectives, this restricted sample size may mean that some viewpoints are underrepresented, potentially limiting the generalizability of the findings for staff-related themes.

## Findings

### Themes from interviews with patients

**Figure 1.**
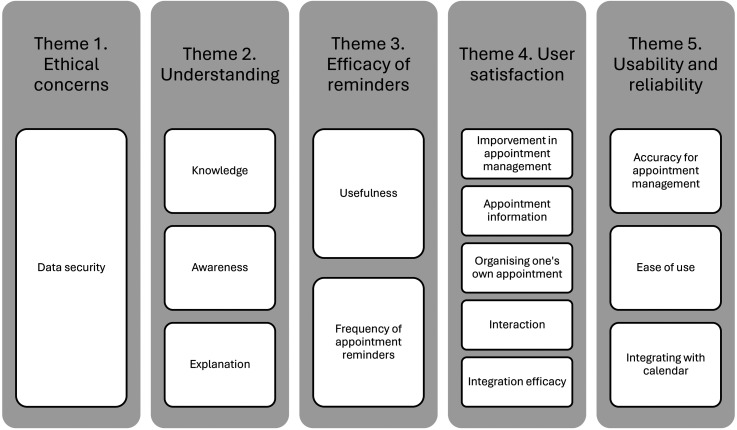
Diagram of qualitative patient themes and sub-themes.

In our sample of 12 patients, nine identified as male and three as female, all were aged over 18 years. Five people were living with cancer, two patients had kidney disease, and five people had eye problems. All had experienced the appointment management technology, but not necessarily the AI aspects. Qualitative data were organized into five themes (see Figure 1) and are now presented with illustrative quotes. See Table 2 for a quantitative overview of responses.

**Table 2.** Quantitative summary of patient themes and sub-themes.

Theme and Sub-Theme	No Concern	Some Concern
1. Ethical concerns		
Data security	10	2
3. Efficacy of reminders		
Usefulness	12	0
Frequency of appointment reminders	6	6
4. User satisfaction		
Improvement in appointment management	10	2
Appointment information	7	3
Organizing one’s own appointment	9	3
Interaction	11	1
Integration efficacy	10	2
5. Usability and reliability		
Accuracy for appointment management	10	2
Ease of use	3	9

Note: Theme 2, Understanding, was excluded from the table because its elements were not meaningfully quantifiable.

### Ethical concerns

#### Data security

All participants spoke about data security and the majority reported not being concerned about medical technology data protection, feeling no more at risk than when using other social media platforms or having their data stored by the hospital. Luke had a highly relaxed attitude towards data privacy, “The security would not be an issue for me, personally. If it was breached: if it went halfway round the world to everybody, it wouldn’t bother me.” Daniel echoes a similar lack of concern, comparing the security of his medical records to that of other systems where users trust that their data will be kept confidential,

I don’t have any concerns. No more concerns than to any other app *(application*) that you sign up to and agree to the terms and conditions and just … You have faith, don’t you, that your details will be kept confidential

One participant showed a sense of resignation and scepticism regarding data security in the modern digital age,

“Well, you know, in this day and age, is anything safe? Because there's always this hacking going on. Is anybody's data safe? But it's the world we live in.”

The few participants who did express concerns felt a general unease about data being lost, which was not specific to the AI system here. Steve noted, “You see on the news where information has been mislaid and people have got it, it's fallen into the wrong hands so I’m a bit wary about that side, but I am all for the technology.” Max added, “It worries me because it doesn’t matter what you do, these hackers seem to be able to get into anything, don’t they?”

The statements reveal varied attitudes towards data protection among participants, however, most were unconcerned, viewing the risks of the AI system as similar to those of social media or other apps. Some were indifferent about who sees their medical records and trusted in the confidentiality promised by the technology providers. Others acknowledged the risk of hacking but accepted it as part of modern life. A minority expressed specific concerns about data loss and breaches in general, highlighting data misplacement and hacking capabilities. Overall, there is general trust in data protection systems but also an awareness of inherent risks, with some expressing caution and scepticism.

The general trust observed may stem from patients’ exposure to data security measures in other digital platforms, such as banking or social media, where breaches are uncommon. However, scepticism and concerns among a minority highlight the need for transparent communication about the security protocols specific to the AI system, such as encryption or compliance with data protection regulations (e.g., General Data Protection Regulation (GDPR)). Providing patients with concise, accessible information about these measures could alleviate concerns. Additionally, establishing a clear protocol for addressing breaches, including patient notification and mitigation plans, could enhance trust further.

### Understanding

#### *Knowledge* about system being used

There was a general lack of knowledge among participants about the system being used to manage their appointments. For example, Max mentioned, “Obviously for the appointments, the artificial intelligence is supposed to be really good but I don’t know anything about it and I’ve no interest in reading up about it.” Similarly, Tom stated, “I don’t know the ins and outs of the system, I don’t know how it's been stored, I don’t know how it's connecting to the NHS backbone.”

Overall, interviews highlighted a lack of knowledge (and motivation for gaining such) regarding the AI appointment management system and its integration with healthcare infrastructure. This likely stems from insufficient communication during the system’s implementation phase. Patients might not have received accessible, engaging information about the system's purpose and capabilities. Offering brief, user-friendly informational videos, infographics, or FAQs at the system's rollout could improve awareness.

#### Awareness

Most patients were unaware of the existence of the s*ystem* responsible for managing their appointments and reminders. Tom commented, “the messages and things that I’ve received, I haven’t even realised which ones of those were from the appointment management system.” Similarly, Mick expressed uncertainty, saying, “I don’t know if I know anything. I mean, I can suppose what it is. Have I received appointments from it? It seems familiar. Do I get text messages from the appointment management system?” Daniel added,

Not a great deal. I have had various appointments sent to me, either by text or through the NHS app or by email. So, I get appointments sent in various different forms. I’m not too much aware of where the (this) system fits into that.

Rio highlighted his lack of awareness, stating, “Well, I didn’t know it was in force for a start, so it's a bit hard to say, isn’t it, really… I just thought it was exactly the same as it's always been.”

These statements indicate a lack of awareness among patients regarding the origin and role of the system in their appointment management, with some expressing familiarity but uncertainty about its specific function. The lack of awareness could be attributed to the system's seamless integration into existing appointment workflows, which, while beneficial, inadvertently obscures its role from patients. Ensuring that appointment notifications clearly indicate they are powered by AI technology and providing brief descriptions of its benefits could enhance awareness. For instance, a tagline such as “Managed by AI technology for your convenience” included in appointment messages could help highlight the system’s involvement.

#### Explanation

Overall, participants felt that the system being used to manage their appointments wasn’t adequately explained to them. For example, Mick mentioned,

I didn’t know it was the AI tech that was texting me. It slipped my mind, looking back. I knew I’d seen it somewhere. Looking back, it's just buried in the http web address. That's the only reference to it. So maybe it could be a little bit more overt so people know that AI are involved.

Steve expressed a desire for more information, stating, “I’d probably have liked to have known more but the time we had talking about it, obviously we didn’t have a lot of time to go really in-depth with it.”

These perspectives collectively underscore the need for enhanced communication strategies to better inform patients about the system, it's functionalities, and its role in their healthcare management, thereby improving transparency and potentially fostering trust in its use. In contrast, these findings potentially highlight a seamless integration of the system into the existing infrastructure. The insufficient communication about the system may result from assumptions that patients would not require or desire detailed explanations. Implementing a structured communication plan that includes patient-facing materials, such as welcome emails or leaflets, could address this gap. These materials should outline the system's role, its benefits, and ways to interact with it effectively. Offering patients the ability to opt-in to receive more detailed explanations could cater to varying preferences.

### Efficacy of appointment reminders

#### Usefulness

All patients emphasized the usefulness of appointment reminders provided by the system. Steve highlighted their practical value, noting,

They are quite variable and obviously you get an appointment in July and it's not till October so you tend to forget you’re going to hospital so getting the text messages a couple of days before, or the day before, you think oh yes, I’ve got that one coming up so I do find text message reminders very helpful.

Mick shared similar sentiments, particularly appreciating the reminders amidst a busy schedule, stating, “In my real life, when I’m teaching etc. etc., it's really useful because it's hectic and busy and a lot of stuff going on. It's useful just to have that reminder in case it's slipped the diary or whatever.” He recalled a specific instance where the reminder prevented him from missing an appointment, saying, “It's excellent, actually. I had one this morning vis-à-vis an appointment next week, which I’d sort of forgotten about.” Luke echoed this, acknowledging the practical benefits of reminders for managing appointments, remarking,

But a reminder is very useful. It's so useful. The doctors’ phone appointment, I quite often forget that – or I have, in the past, forgotten it. So it's quite nice having a reminder: ‘Oh, you’ve got a phone appointment in two days’ time.’ It's quite nice.

These highlight the crucial role of appointment reminders being useful in helping patients stay organized and prepared for their medical appointments, highlighting their effectiveness in mitigating forgetfulness and ensuring timely attendance. The usefulness of reminders likely stems from the growing reliance on mobile technology for managing daily tasks. However, as highlighted further down, the effectiveness of these reminders could be further enhanced by personalizing the content and delivery. For example, patients could choose preferred reminder timings or channels (e.g., text, email, or app notifications). Including brief details about the appointment, such as its purpose or location, could make reminders even more effective.

#### Frequency of appointment reminders

Half of the participants expressed satisfaction with the frequency of appointment reminders they received. Mick noted,

I think it's probably about right. I'm being honest: I’m struggling to remember if I’ll get another reminder. So I got this appointment today – it's a week today – so exactly a week in advance, I got a text. I’m trying to think if I will get another text or not. But it's certainly not too much, no. It's not annoying.

Daniel shared this sentiment, saying, “Yes, so when I get a text message it's good that I get reminded of that. There are not too many. I don’t think you can have too many.” Steve also agreed, stating,

No, I think I just get the right amount because I do have quite a lot of appointments with doctors and hospitals, so they are quite valuable to me because you get that many, you tend to think what have I got this week.

Apart from one participant who desired more reminders, some felt there were too many, with Luke describing receiving them via different modes as wasteful, “No. To me, I just think it's wasteful when it comes through both on email and text. One would suffice for me on the occasion.” Robert echoed similar sentiments, saying, “Sometimes, on reminders, I’ve found it – in my case: maybe not for other people – probably a little excessive.” Matthew expressed similar, stating, “And then I get reminders and so on: usually far more reminders than I need but … Occasionally, I also get a letter through the post.”

The mixed feedback on reminder frequency may reflect individual differences in preferences and communication styles. Allowing patients to customize the frequency and mode of reminders, such as opting out of receiving multiple notifications via different channels, could reduce dissatisfaction. Additionally, a periodic review of reminder settings to match patient preferences could help balance the volume of notifications.

### User satisfaction

#### Improvement in appointment management

Since the introduction of the system, all patients described no improvement in their appointment management and scheduling. For example, when asked about improvement, Mick stated, “No, it's been fine: but no difference, is the truth…It certainly didn’t get any worse.” Similarly, Rio commented, “…they’re about the same as far as I can see. I can’t see any improvement.”

The lack of perceived improvement may stem from limited visibility into how the system enhances processes. Patients may not notice behind-the-scenes efficiencies, such as reduced DNA rates, if these do not directly impact their experience. To address this, healthcare providers could share insights about the system's overall performance, such as metrics showing improvements in clinic efficiency or reduced appointment wait times. Engaging patients in feedback sessions could also help identify areas where visible improvements are needed.

#### Appointment information

A few patients expressed dissatisfaction with the level of detail provided in their appointment scheduling messages, particularly regarding the location of the appointment. Mick noted, “Sometimes it would have been useful, in say the text message, if it had an embedded link so that you can click on it and it tells you the department, so there are features there that I feel could be added that would be more useful.” He further elaborated on his suggestion, saying, “You could click on a link and it tells you here is your appointment, here's who it's with, here is the department and also here is a little map showing you where it is.” Tom also highlighted the lack of information provided, stating,

One of the text systems just literally tells me that I’ve got an appointment coming up and there's no other information, but some of the texts I’ve received have told me you have an appointment at Queen's Medical Centre at this time, but it doesn’t actually tell me what the appointment is for.

The dissatisfaction with appointment information may result from limitations in message templates or system design. To address this, embedding interactive links in appointment messages could provide additional details, such as the department, clinician's name, and a map of the clinic location. These enhancements could make the information more actionable and reduce patient confusion.

#### Organizing one's own appointments

Most patients found the appointment management system useful for organizing and tracking their appointments. Daniel highlighted the convenience of having appointments accessible on mobile devices, stating,

We’re all tied to our mobiles, aren’t we? And I think having it available to refer to and to be able to put in your calendar is something that has got much better than having a letter, which could get lost in the post – or a telephone call, which you could forget. So, having it done in that way is much better, yes.

Max emphasized the system's benefit for managing memory challenges during medical treatments, saying,

One of the problems I suffered with in particular when I was having the chemo, and still to a certain degree now, is I have got a very bad memory, certainly I would have to constantly look at the letter because I would forget when the appointment was for and of course you have always got your phone with you so it's very easy just to flick on the phone and it's always there.

A recurring issue reported by many patients is the system's limitation to viewing only future appointments. They expressed a desire to access past appointment records as well. Matthew noted, “If I go to ‘appointments’ …I don’t see past ones: I just see futures.” Luke similarly observed, “The system seems to run only on what's going to happen currently or in the future. Current or future or ‘pending’ – whatever the right word is.” Matthew elaborated on the importance of accessing past appointments for medical history tracking, stating, “Tracking things back and someone says, ‘When was your last MRI?’ things like that, it's still very difficult to do.”

The limitation to future appointments may result from system design choices that prioritize immediate scheduling needs over comprehensive historical access. Expanding the system to include a searchable archive of past appointments could significantly enhance its utility. This feature could help patients manage their medical history more effectively and reduce reliance on paper records or memory. Additionally, integrating this functionality with other medical records could further streamline patient experience.

#### Interaction

Interaction with the technology emerged as a significant source of frustration for most patients who had interacted with it. They expressed a strong desire for the ability to actively manage their appointments directly via the technology, including options to accept, decline, or reschedule appointments. Daniel pointed out the current limitations, stating, “It's if I need to cancel or make changes: it just says, ‘The hospital has not provided a phone number for this appointment. Please refer to any letters you have received.’ So I would still have to go back to manually looking at the letter if I needed to make a change.” He emphasized, “If I needed to change it or if I wanted to speak to somebody … So, in terms of one-way information to me, it's great: two-way, not so great. Apologies if that sounds harsh.” James echoed these frustrations, highlighting the potential consequences of difficulty in managing appointments, saying,

The more difficult it is to rearrange appointments, the more difficult it is to get through to a department via the phone and the more chance there is of missed appointments because people are just going to get annoyed and not do it, or get busy and stressed.

Max shared his expectations for future functionality, stating, “I’m hoping in the future you will just be able to tick a box saying I can’t make this appointment and it should either give you an option to reschedule, if it can do that, and if not it should have a call-back feature where someone rings you to give you a new one.” Luke highlighted his current passive interaction with the technology, emphasizing, “Like I said earlier, I just receive information. I’m not manipulating the system: I’m not making appointments; I’m not talking to people and what-have-you…at the moment, I’m just receiving, ‘Here you go. Here's your appointment letter.’” Tom pointed out specific issues with accessing detailed appointment information, stating, “There's no way to find the macular clinic contact number through the *technology*. It just tells me there's an appointment, and a lot of the time I don’t know what the appointment is for and it doesn’t necessarily give the department or the contact details, so I’ve had to go fishing around.”

The lack of interactivity likely stems from system design constraints that prioritize basic communication over active patient engagement. To address this, incorporating interactive features such as appointment rescheduling, cancellations, and direct messaging with clinics could significantly improve user experience. Features like callback requests or automated rescheduling options could further reduce patient frustration and missed appointments.

#### Integration efficiency

Streamlining appointments and integrating systems emerged as a significant topic across the interviews. The majority of participants highlighted frustrations with the technology's inability to consolidate all their appointments from various sources into one unified platform. Max noted discrepancies, saying, “Some of the appointments coming from the consultants come different to some of this technology things so you don’t get everything on the app.” Tom echoed confusion over multiple systems, stating, “I did receive a text from one system and then a text from a different number, so there was definitely some sort of hospital system reminding me about an appointment and then a different system which I assume was this technology.” Tom further compared the lack of integration to seamless tech ecosystems like Apple products, remarking, “Everybody knows if you buy an Apple phone it all links together with other Apple products so it all connects, and we’re not really seeing that with this system.” Jarad expressed a desire for a more centralized approach, suggesting, “Maybe, if it tied in with this *PatientView* or whatever it's called… It would be better if it would just be a bit more centralized, I suppose.” Matthew highlighted the proliferation of separate healthcare apps, stating, “Why have yet another? NHS apps: I’ve got NHS; I’ve got this AI technology and, to deal with a lot of things, I’ve got *SystemOnline* and there's another one.” Sam shared his confusion with the overlapping systems, explaining, “Well, it is (confusing) because, as I say, in hospital, you use this technology for appointment, but I had to get my blood results and that comes through *Doctor Knows Best*” (another technology).

The fragmentation of healthcare systems likely arises from the use of multiple technologies developed independently. To streamline patient experience, consolidating data across platforms into a single interface, such as an integrated patient portal, could reduce confusion. Interoperability standards, supported by collaboration between developers and healthcare providers, could facilitate this integration.

### Usability and reliability

#### Accuracy for appointment management

Most patients expressed satisfaction with the accuracy of their appointment management. Tom affirmed, “Yes, it was definitely accurate, I didn’t receive any appointments that didn’t exist or that were late or misrepresented.” Similarly, Luke stated, “I haven’t had any problems with it in any way, shape or form. It's fine.” Matthew emphasized, “It's always been correct. I’ve never been sent a random appointment: it's always been directed to me and, yes, I’ve never had anything that's been wrong about it.” Robert added, “Very well, actually, to be quite honest with you. I’ve never had any particular occasions where I’ve been put out and I’ve had to make a wasted journey or anything.”

However, a few patients did report specific issues with appointment accuracy showing on the app. Tom noted, “I’ve got an appointment for urology in, I think, January or February, and that's not on.” Sam mentioned, “Last time I was in the transplant clinic, I made an appointment to see the transplant nurse in two months and then the renal doctors in four months. And it's only the transplant nurse's appointment that's showing.” Matthew highlighted,

Some appointments seem to miss the system altogether. So I had a CT scan on Friday that was the result of a phone call on Tuesday and never, ever appeared in the appointment list anywhere. And no letters, no nothing. But I went: it was there; it was booked. So, the usage seems to me to be very inconsistent.

These instances indicate a general satisfaction with appointment accuracy among most patients, tempered by sporadic issues that highlight the need for improved consistency and reliability in the system. The reported inaccuracies may result from discrepancies in data synchronization between different hospital systems or incomplete integration with appointment scheduling software. To address this, implementing real-time data updates and ensuring all appointments are captured across systems could reduce such inconsistencies. Training staff to verify appointments and routinely audit the system could further enhance reliability. Additionally, providing patients with a feature to report missing or incorrect appointments could help identify gaps in the system.

#### Ease of use

Issues related to the usability of the system were prevalent across the interviews, highlighting various challenges faced by patients. Daniel found logging into relatively straightforward, noting, “So your log in is quite easy because it's just your name, date of birth and your postcode.” However, he acknowledged recent changes requiring a security code, which he described as generally secure but potentially cumbersome. Conversely, Matthew expressed frustration with the login process, stating, “It assumes that I don’t have a login: that I need to … because, I’ve got to press an extra step to continue to login.” He found the process confusing and disjointed, further explaining, “It doesn’t ask for the password on the same screen. And it then wants the password and I put that in. It just gets very confusing.” Tom echoed usability concerns, particularly regarding accessing appointment information. He criticized the system for its inefficiency, saying, “What's quite an irritating issue is that every single time you have to enter your date of birth, your postcode and all this other stuff, it's quite annoying.” He emphasized the lack of simplicity in accessing PDFs and navigating the system.

One participant, Daniel, described the technology as intuitive, attributing its ease of use to his familiarity with technology. He remarked, “I think it's intuitive. I don’t think it's that complicated to get from being informed from an email or whatever and then asked to click on the link to go and view your letter or appointment or whatever.”

Vision impairment posed additional challenges for some patients. Rio highlighted difficulties due to restricted vision, stating, “No, I can’t do it online. I’ve got restricted vision as well.” Tom, anticipating potential vision loss due to macular degeneration, expressed a desire for accessibility features like text size adjustment, which he found lacking in the AI technology.

Overall, while some patients found aspects of the system user-friendly, several usability issues such as complex login procedures, inefficiencies in accessing information, and lack of accessibility features for vision impairment were identified, suggesting room for improvement in enhancing user experience and accessibility. The usability challenges may stem from design decisions that prioritize security over user convenience. Simplifying login processes by integrating single sign-in options and offering user-friendly guides for navigation could alleviate frustrations. Incorporating accessibility features, such as adjustable font sizes or screen reader compatibility, would make the system more inclusive for patients with vision impairments. Regular feedback loops from patients could help identify and address persistent usability issues.

#### Integrating with calendar

Several patients expressed a desire for the system to integrate with their personal calendars. Mick mentioned, “it would be interesting, possibly, being able to link it with one's own calendar – Google calendar – and whatever else. I think there would be some nervousness from a lot of people about that.” Daniel emphasized the convenience, saying, “Because I have other than work things that come through with the ability to ‘add to calendar’ and I just add it, then it's the second line for me, to know that I’ve got an appointment booked, rather than rely on a reminder. If it didn't, it would be good to have.” Tom suggested enhanced functionality, stating,

it could say you have an appointment at this time, would you like me to put that in your Google, Apple, Outlook calendar for you, yes or no, or if you’ve got an app and don’t need that would you like me to set a reminder for you, would you like me to remind you an hour before your appointment.

These comments reflect a common desire among patients for the system to seamlessly integrate with their existing digital calendars, providing them with proactive options to manage their appointments more efficiently and reduce reliance on separate reminders. The lack of calendar integration likely reflects a limitation in the system's functionality rather than a technical challenge, as such features are common in many applications. Developing a simple “add to calendar” feature compatible with popular platforms like Google Calendar, Apple Calendar, and Outlook could significantly improve usability. Offering customization options, such as setting reminders directly within the calendar integration, could further enhance the patient experience.

#### Themes from interviews with staff

In our sample of seven staff members interviewed, all identified as female, aged over 18 years. This included one project manager for the AI technology, one clinician, two clinic managers, two administrators, and one person responsible for calling patients at risk of non-attendance for appointment. All had direct experience of the AI technology in practice.

Qualitative data were organized into five themes: understanding and hesitancy, barriers and drivers, experience using the AI technology, appointment management, and sustainability. See Table 3 for a quantitative overview of responses.

**Table 3.** Quantitative summary of staff themes.

Theme	No Concern	Some Concern
Understanding and hesitancy	5	2
Appointment management	4	3
Sustainability	1	6

Note: Themes related to Experience using the AI technology and Barriers and drivers were excluded as they were not meaningfully quantifiable.

### Understanding and hesitancy

Overall, there was good understanding from staff of the potential usefulness of AI in healthcare, with few concerns about data protection in AI use generally. One interviewee said, “Yes, I do trust it, but obviously I still think it's only as good as the data you’ve put in.” Only one staff member was concerned about data protection, saying, “Yes, it worries, me, it's always there, I work in IT and so it's a worry, but I wouldn’t say it's as much of a worry as missing patients with terminal illnesses or anything like that.”

There was also concern about the ethics of using a system that predicted those who might not attend for appointments, with one staff member saying, “So for me, ethically there's loads of questions about are we making assumptions of people based on their postcode, based on their colour, based on their age, and that could be concerning.”

Concerns about the ethical implications may stem from a lack of transparency in how AI predictions are generated. Staff may be uncertain about whether the models incorporate potentially biased variables, such as demographics or socioeconomic status. To address this, clear documentation and training on the AI system's algorithms, highlighting safeguards against bias, could build trust among staff. Additionally, implementing oversight mechanisms, such as periodic audits of the AI model for bias, could reassure staff of its ethical integrity.

### Barriers and drivers

Further thoughts about the quality of data used for AI technology were highlighted when staff asked specifically about the appointment management technology. “I have noticed that accuracy-wise there are some names that are always on there or there quite frequently, so I think accuracy-wise it probably does show the people that are more likely, if they are already on there.”

However, trust in the quality of the data used to predict non-attendance appeared to be increasing over time,

The model itself is retrained once a week automatically so it's constantly bringing in the new data from new appointments, it's constantly refreshing itself which we didn’t have initially, so we think we’ve only had that for the past couple of months and that's definitely has become more robust because we’re able to bring in that data more regularly.

One potential barrier was using the appointment management technology across specialities (learning from one speciality might not suit another). “It's okay from one Trust (hospital) to another, but it's about the specialties within the Trust (hospital), so what might work in nephrology might not work in ophthalmology.”

Barriers related to data quality and variability across specialities may stem from the AI system being trained on limited or inconsistent datasets. To address this, ensuring that the system is trained on a diverse dataset encompassing a range of specialities could improve its applicability and accuracy. Additionally, engaging staff from different departments in co-designing workflows could ensure that the system meets the specific needs of various clinical contexts.

### Experience using AI technology

There was mixed response to the experience of using the appointment management technology. One general issue is that some patients block texts from unknown numbers, “When a text is generated, we can view the technology to see if it has been sent and sometimes it says ‘Failed.’ I presume that the patients have got a block on the number.” One clinical team highlighted that the DNA prediction tool did not have all the necessary information needed to make an efficient call to the patient,

…when you're ringing through the dashboard you can say you’re coming to this clinic on Tuesday 14th at this time, [and the patient says] could you tell me which site it's at please, no, because it's not there. So what they [the callers] have to do is go back into it [the hospital system] and find out where that clinic is being held on that day.

Another issue was the response of patients when called by the confirmation caller.

Some people seemed quite offended by me calling and double checking, I don’t think they had ever DNA-ed an appointment before which made it quite an uncomfortable conversation if you were having to try and explain that it's nothing on your behalf, we’re just calling to double check.

Issues with blocked texts may reflect patient concerns about spam or a lack of familiarity with the sender. Addressing this could involve branding the messages clearly as being from the hospital, along with a brief explanatory note. The lack of detailed information in the DNA prediction tool could stem from incomplete integration with hospital systems; improving interoperability between the AI dashboard and other systems could streamline communication.

### Appointment management

There was a mixed response regarding the impact of the AI technology on appointment management, though the majority expressed no concerns about its capabilities in managing appointments effectively. One speciality was more positive than the other,

[Name] has continued to do the confirmation calling for the clinics that we’ve identified and we looked at numbers, we definitely have seen a decrease in DNAs…but if we’re going to phone these patients, we need to be doing it a week in advance and that gives us time to fill the clinics.

One main consideration was how easy it would be to fill the appointment,

I don't know how much difference the ringing is making, if I’m honest …by the time we ring them and they say they are not going to come, it's almost too late to fill that slot for a follow-up clinic.

The variability in perceived impact may arise from differences in clinic workflows or patient demographics. Developing clinic-specific strategies, such as varying the timing of confirmation calls, could optimize the AI system's effectiveness. Additionally, monitoring DNA rates over time and sharing success stories across departments could motivate wider adoption.

### Sustainability

There were general concerns about whether out-patient departments would continue to use the technology unless they saw meaningful outcomes. One interviewee said,

The thing I would say about this (AI) model is if it is going to be worth doing, there has to be a meaningful change in the DNA rates, and I think a meaningful change in the DNA rates is 10% as a minimum.

Another person said,

I worry that we’ll see a reduction in DNAs and claim that that means it's all great but actually it is not a meaningful, clinically relevant reduction in DNAs.

Concerns about sustainability may stem from insufficient visibility into the AI system's long-term benefits. Regular reporting of performance metrics, such as cost savings or improved efficiency, could help justify continued use. Additionally, setting realistic benchmarks for success, aligned with clinical priorities, could ensure that expectations are met.

## Discussion

The insights gathered from the interviews revealed several significant implications for healthcare providers and technology developers alike. First, the varied attitudes towards data protection underscore the critical importance of robust security measures within healthcare technologies. While many users trust in the technology's confidentiality promises akin to social media platforms, similar to other studies, concerns about hacking and data breaches highlight the ongoing need for stringent security protocols to safeguard patient information and maintain trust.^17,18^ These findings align with research on international implementations of AI technologies, such as in the USA and Europe, where privacy concerns are a significant barrier to adoption.^6,19^ Strategies used abroad, such as transparent data use policies, enhanced encryption methods, and public awareness campaigns, could be adopted to build trust in the NHS. Proactively educating patients and staff about these measures and providing clear responses to security concerns could mitigate apprehension and promote acceptance. Data security concerns, if not adequately addressed, have the potential to hinder broader adoption of such AI technologies in healthcare. Strategies to mitigate these concerns should focus on enhancing transparency about data handling practices, implementing state-of-the-art encryption, and ensuring compliance with data protection regulations such as the GDPR. Moreover, fostering trust among patients and staff can be achieved by offering clear, accessible information about the measures in place to secure data, such as anonymization protocols, regular system audits (i.e., evaluation of the system's processes, infrastructure, and security measures to ensure compliance with established standards, detect vulnerabilities, and improve overall data security), and breach response plans.

Another strategy to build trust is to involve end-users, both patients and staff, in the design and implementation phases of the technology. Co-design workshops or focus groups could help developers address user concerns early, ensuring that systems align with user expectations and requirements. Additionally, establishing ongoing communication channels, such as FAQs, dedicated support teams, or user education sessions, can help address questions and reduce apprehensions about data security. Finally, incorporating visible security features that allow users to track how their data is used, may further enhance confidence in the technology.

Moreover, the feedback on appointment reminders indicates a clear preference for personalized communication strategies. Users appreciate timely reminders but express divergent views on their frequency, suggesting a need for customizable settings to better meet individual preferences and streamline appointment management. This echoes findings where tailored communication strategies have been shown to significantly reduce missed appointments and improve patient satisfaction.^3,10^ Previous research suggests no earlier than 7 days, giving patients sufficient time to cancel or reschedule,^20^ but the current findings suggest this preference varies between patients. Integrating international best practices for patient-centred communication, such as options for language preferences and multi-platform delivery (e.g., email, app notifications), could enhance patient engagement.

Enhancing the clarity and informativeness of appointment messages, potentially with integrated details and links (e.g., to cancel or reschedule appointments), could further improve user convenience and reduce missed appointments. This supports earlier findings that healthcare technologies often pay limited attention to the cancellation and rescheduling process.^12^ In systems like MyChart in the USA, interactive features enabling easy cancellations and real-time rescheduling have demonstrated improved appointment adherence and patient autonomy.^21^ Using AI to generate content that adapts to different situations and user needs in real time can help provide more relevant and helpful information.

Usability emerged as another significant area for improvement, with users citing challenges such as complex login procedures and inefficiencies in accessing information. Simplifying login processes, improving navigation, and incorporating accessibility features are essential steps to enhance the technology's usability and ensure it meets the needs of all users, including those with disabilities or technical limitations. Internationally, healthcare technologies such as Germany's DIGA-certified apps emphasize user-friendly interfaces and inclusive design (i.e., intentionally designed to be accessible and usable by as many people as possible, regardless of their individual abilities, disabilities, or technical limitations), demonstrating the potential for scalable solutions that cater to diverse populations.^22^ It is possible that patients are directly comparing their experience of this medical technology with the best sales apps dominating the consumer market. Typically, the latter survive in the marketplace by reducing unnecessary user steps to the greatest extent possible, for instance, minimizing login steps or eliminating clicks from basket to checkout completion. Adapting these principles, while maintaining regulatory compliance, is a challenge that developers must address to meet patient expectations.

The frustration with fragmented healthcare management systems highlights the importance (and potential benefit) of integrating appointment and health information platforms. Enhancing interoperability with existing digital calendars and healthcare systems could streamline administrative tasks, improve efficiency, and ultimately enhance patient satisfaction.^23^ Globally, countries like Denmark and Estonia have set benchmarks in interoperability through centralized e-health platforms, which consolidate patient data and allow seamless communication between systems.^19,24^ Adopting similar approaches within the NHS could alleviate fragmentation concerns while improving user experience.

Continuous improvement is crucial, with user feedback suggesting ongoing iterations to address concerns promptly and enhance the technology's functionality and user experience. Transparent communication about the functionalities, data handling practices, and security measures is vital to build and maintain trust among users (staff and patients) and promote widespread adoption in healthcare settings.^14^ Building trust requires a clear, consistent dialogue with end-users, as evidenced by successful initiatives internationally, where public trust in AI-driven healthcare has been bolstered by active stakeholder engagement and transparency campaigns.^12,24^

In conclusion, addressing these implications comprehensively, by prioritizing security, enhancing communication and usability, improving integration, and fostering transparency, should significantly enhance the effectiveness and acceptance of the technology. By incorporating lessons from international implementations and aligning with global best practices, developers and policymakers can ensure that such AI technologies contribute to better patient outcomes and the broader advancement of digital healthcare services.

### Limitations

First, the study focused on only three specific medical specialties, which may not fully represent the diverse range of healthcare practices and patient needs across all specialties. This scope limits the applicability of the findings to broader healthcare contexts where different specialties may have unique requirements. Additionally, the qualitative elements of the study were conducted at a single NHS site located in central England. This regional restriction means that the findings may not adequately reflect the diversity of healthcare settings, patient demographics, and regional healthcare policies across the entire nation. The limited geographic focus also impacts the full spectrum of demographic characteristics and socioeconomic backgrounds that can influence attitudes towards digital healthcare tools.

Moreover, some patients interviewed had limited awareness or understanding of the technology, which may have constrained the depth of exploration. These factors introduce the potential for selection bias, as participants from certain specialties or regions may not fully represent the broader population of NHS patients and staff. Similarly, response bias may have occurred if participants with stronger opinions (positive or negative) were more likely to engage with the study.

The qualitative elements of the study focusing on acceptability were complemented by other evaluation questions concerning accuracy, safety, effectiveness, and value. Overall, the AI technology appeared to be accurate in terms of DNA prediction and was safe and mostly acceptable to patients. The technology did not adversely affect patients’ mental health or physical wellbeing (including self-reported adverse events) yet did not improve patients’ satisfaction with appointment management. The use of the AI technology did not result in a detectable positive change in DNA rates in three specialties in two NHS Trusts. Leaving a message and/or sending an additional SMS to those likely to DNA was not effective. Talking to people directly to remind them of their appointments did not have a statistically significant impact but had more encouraging results in a very small sample.

Finally, while the insights gained are valuable within the studied context, caution should be exercised when applying them to other regions or healthcare settings without considering these contextual limitations. The full evaluation report will be available later in 2024 (https://researchportal.lsbu.ac.uk/ws/portalfiles/portal/8681929/AI_Award_Final_Report_Template_LSBU_DrDoctor_Final_03042024.pdf).

## Conclusion

In conclusion, this is one of the first published papers that explores patient and staff perceptions of an AI technology that aims to optimize out-patient management in the NHS in the UK. Insights gleaned from participants’ interviews highlight both strengths and areas for improvement in healthcare management technology. While attitudes towards data security varied widely, ranging from casual acceptance to specific concerns about privacy breaches, there is an overarching trust in the system's ability to safeguard personal information, albeit with reservations. The divided opinions on appointment reminders underscore the importance of tailored communication strategies to meet diverse patient preferences effectively. Addressing concerns about message clarity and interactive functionalities is crucial for enhancing user experience and ensuring the technology meets the needs of all users, including those with accessibility challenges. Furthermore, the desire for seamless integration with digital calendars reflects a growing expectation for technology to streamline healthcare administration.

These findings have practical implications for NHS policymakers as they consider future strategies for implementing AI technologies in outpatient care. Tailored communication strategies, enhanced usability, and integration with existing healthcare systems should form the foundation of these strategies. Policymakers should also prioritize initiatives that address data security concerns and transparently communicate the safeguards in place to build trust among both staff and patients.

For healthcare providers, the results suggest the need for ongoing training and education to ensure staff are well-equipped to use these technologies effectively. Future implementation efforts should involve iterative feedback loops with end-users to refine the technology and improve acceptability. Technology developers should focus on creating user-friendly systems that incorporate accessibility features and offer customizable functionalities, such as personalized reminder settings and interactive scheduling options.

Future research should explore the scalability of these findings across different specialties and geographic regions, as well as investigate the long-term impacts of AI technologies on DNA rates, patient satisfaction, and operational efficiency. Collaborative efforts between researchers, policymakers, and technology developers will be crucial to ensuring that AI solutions are both effective and equitable, meeting the diverse needs of the NHS patient population.

These findings underscore the ongoing need for iterative improvements in technology design and communication practices to foster transparency, user satisfaction, and trust in digital AI healthcare solutions. By addressing these priorities, the NHS and technology developers can work together to create more effective, user-centred systems that enhance the quality of outpatient care.

## Supplemental Material

sj-pdf-1-dhj-10.1177_20552076251321016 - Supplemental material for Artificial intelligence machine learning-driven outpatient appointment management: A qualitative study on acceptability Supplemental material, sj-pdf-1-dhj-10.1177_20552076251321016 for Artificial intelligence machine learning-driven outpatient appointment management: A qualitative study on acceptability by Kerry V. Wood, Daniel Frings, Chris Flood and Nicola Thomas in DIGITAL HEALTH
